# Human Dental Pulp Stem Cells Differentiate into Cementoid-Like-Secreting Cells on Decellularized Teeth Scaffolds

**DOI:** 10.3390/ijms232415588

**Published:** 2022-12-09

**Authors:** Manuel Mata, Santiago Peydró, José Javier Martín de Llano, María Sancho-Tello, Carmen Carda

**Affiliations:** 1 Department of Pathology, Faculty of Medicine and Dentistry, Universitat de València, 46010 Valencia, Spain; 2 INCLIVA Biomedical Research Institute, 46010 Valencia, Spain; 3 Biomedical Research Networking Center of Respiratory Diseases (CIBERES), 28029 Madrid, Spain; 4 Facultad de Odontología, Universidad Europea de Valencia, Passeig de l’Albereda, 7, 46010 València, Spain; 5 Biomedical Research Networking Center on Bioengineering, Biomaterials and Nanomedicina (CIBER-BBN), 28029 Madrid, Spain

**Keywords:** cementum, cementogenesis, human dental pulp stem cells (hDPSCs), dentin, dentin sialophosphoprotein (DSPP), microtopography

## Abstract

Periodontitis is a common inflammatory disease that in some cases can cause tooth loss. Cementum is a mineralized tissue that forms part of the insertion periodontium and serves to fix the teeth to the alveolar bone. In addition, it acts as a reservoir of different growth and differentiation factors, which regulate the biology of the teeth. Cementogenesis is a complex process that is still under investigation and involves different factors, including dentin sialophosphoprotein (DSPP). In this work we studied the role of surface microtopography in the differentiation of human dental pulp stem cells (hDPSCs) into cementoid-like secreting cells. We cultured hDPSCs on decellularized dental scaffolds on either dentin or cementum surfaces. Cell morphology was evaluated by light and electron microscopy. We also evaluated the DSPP expression by immunohistochemistry. The hDPSCs that was cultured on surfaces with accessible dentinal tubules acquired an odontoblastic phenotype and emitted characteristic processes within the dentinal tubules. These cells synthesized the matrix components of a characteristic reticular connective tissue, with fine collagen fibers and DSPP deposits. The hDPSCs that was cultured on cementum surfaces generated a well-organized tissue consisting of layers of secretory cells and dense fibrous connective tissue with thick bundles of collagen fibers perpendicular to the scaffold surface. Intra- and intercellular deposits of DSPP were also observed. The results presented here reinforce the potential for hDPSCs to differentiate in vitro into cells that secrete a cementoid-like matrix in response to the physical stimuli related to the microtopography of contact surfaces. We also highlight the role of DSPP as a component of the newly formed matrix.

## 1. Introduction

Different factors including infection, trauma, or orthodontic tooth movement, as well as others, can contribute to the chronic inflammation of the tissues surrounding the teeth and consequent periodontal disease, which is characterized by the destruction of periodontal tissues, including cementum, periodontal ligament, and alveolar bone, leading, if not treated properly, to the loss of affected teeth [1]. 

Periodontitis treatment has relied on the complete scaling of the subgingival and supragingival dental tissues, with diligent maintenance between regular cleanings in the dental clinic [2]. Although this treatment is effective, it unfortunately does not cure periodontitis, it only slows it down. Therefore, complete regeneration of the periodontium has not yet been achieved [2]. 

With the purpose of achieving the regeneration of periodontal tissues, numerous studies have been published from the perspective of tissue engineering. Different materials have been tested including, but not limited to, chitosan, nanoparticle-based scaffolds, and calcium phosphate-based grafts including hydroxyapatite or calcium phosphate cements. In addition, various growth and differentiation factors such as TGF, BMPs, or FGF have been used, as well as stem cells with different origins. Unfortunately, the results obtained cannot be extrapolated to patients with periodontitis [3,4,5,6,7].

Cementum is the mineralized tissue that covers the roots of teeth and serves to attach the tooth to the alveolar bone through the collagen fibers of the periodontal ligament. However, this definition of cementum is somewhat simplistic, as it does not consider many of its functions in relation to periodontal regeneration. Cementum is a complex matrix that acts as a reservoir for growth and differentiation factors such as PDGF, ILGF, TGF-beta, and different BMPS, which are produced during the formation of cementum, stored in the cementum matrix, and released gradually when they are needed, acting as organizers of the regeneration of the periodontium [8]. Under physiological conditions, new cementum is deposited on the tooth root and acts as a barrier to epithelial growth and microbial colonization, and hence defects in the deposit can lead to periodontal disease [9,10]. 

The organic components of adult cementum are secreted mainly by cementoblasts, which are cells derived from mesoderm and responsible for cementum maintenance and regeneration [11]. There is evidence showing that certain dental mesenchymal stem cells, including periodontal ligament stem cells (PLSCs), apical derived stem cells (ADSCs), or dental follicle stem cells (DFSCs), can directly differentiate directly into cementoblasts [12,13,14,15,16]. Human dental pulp stem cells (hDPSCs) are self-renewing mesenchymal stem cells (MSCs) that reside within the perivascular niche of the dental pulp [13,17,18], which are thought to originate from the cranial neural crest, which express both MSCs and neural stem cell markers [19]. The hDPSCs are readily obtained from extracted third molars for orthodontic purposes, and under specific conditions, can differentiate in vitro into a variety of cell types, including neurons, odontoblasts, osteoblasts, adipocytes, and chondrocytes [20,21]. Nevertheless, their ability to differentiate into cementoblasts remains under investigation. 

The relative success of the different biomaterials used for periodontal regeneration has prompted the use of new therapeutic options, such as the use of dental mesenchymal stem cells [22,23]. The differentiation of MSCs into cementoblasts is a complex process that requires several biochemical factors that are part of the tissue microenvironment [24]. However, such a tissue microenvironment comprises different elements including not only biochemical factors but also other elements such as collagen, elastic fibers, and ground substance components, as well as physical forces. We recently demonstrated the effect of cell-interacting surface topography on the differentiation of MSCs into odontoblasts, using a cell-free dentin scaffold [25].

Dentin sialophosphoprotein (DSPP) is a key protein in the control of dentinogenesis. This protein has been detected by immunohistochemistry in odontoblasts (from immature to secretory), dentin, dentinal tubules, dental pulp, and preameloblasts [26,27,28,29,30,31]. DSPP has also been detected in cementoblasts and the alveolar bone in animal models [32]. Nevertheless, its expression and role in human cementogenesis remains under investigation.

The objective of this work was to evaluate the potential of hDPSCs to differentiate into cementoid-secreting cells when cultured on scaffolds obtained from decellularized teeth. The role of scaffold microtopography in inducing hDPSCs to a secretory phenotype, and in the cellular expression of DSPP, was investigated.

## 2. Results

### 2.1. Cell Morphology and Extracellular Matrix Characteristics of hDPSCs Cultured on Surface A (SA)

**Figure 1:**
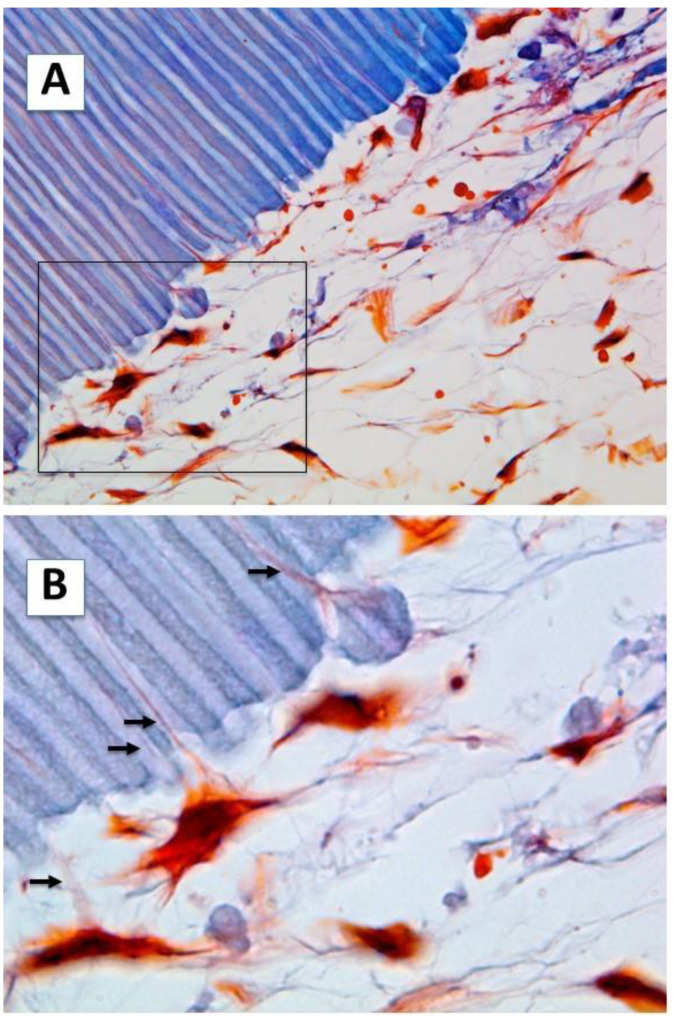

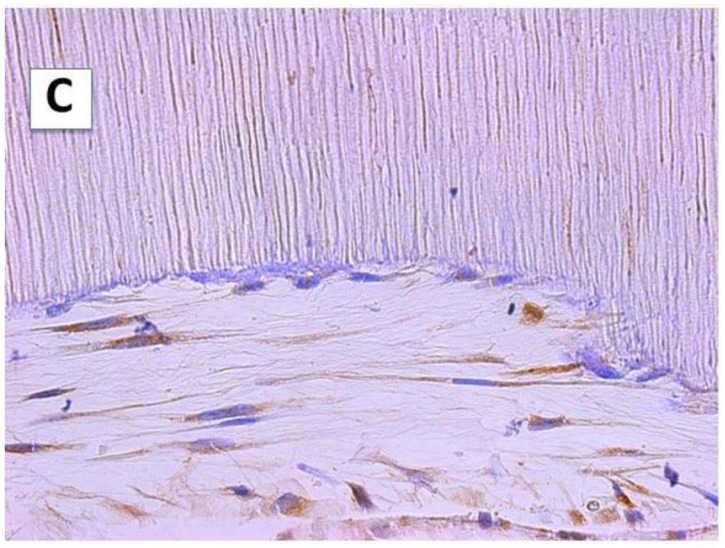
The hDPSCs were cultured on the surface A (SA) of the scaffold for 6 weeks and processed by light microscopy. (**A**) The hDPSCs adopted an odontoblastic morphology, emitting processes within the dentinal tubules; the cells were surrounded by a reticular-like intercellular matrix with fine collagen fibers. Masson’s trichrome staining (40×). (**B**) Close-up of the area delimited by the square in (**A**). On the dentin surface with the tubules perpendicular to the surface, the extracellular matrix was low in collagen and some hDPSCs emitted several processes (arrows) within the dentinal tubules (100×). (**C**) Immunohistochemical analysis of the hDPSCs cultured on the SA. The cells showed intracellular DSPP deposits, both in the cell body and in the cellular processes located within the dentinal tubules (40×).

Characteristic reticular connective tissue was observed, with fine collage fibers and deposits of dentin sialophosphoprotein (DSPP) on the surfaces of the scaffolds with accessible dentinal tubules (Figure 1). The hDPSCs acquired an odontoblast-like morphology, which are characterized by a flat shape and growth that is perpendicular to the dentin scaffold surface. Several cellular processes were observed within the dentin tubules (Figure 1, panel B). The cells presented a stellate morphology, with little cohesion between them, developing cytoplasmic extensions as an odontoblastic process towards the dentinal tubules, and in some cases, several extensions were visible in the same cell. These odontoblast-like cells were positive for DSPP, which was also detected in the cell processes occupying the dentin tubules (Figure 1, panel C). However, DSPP deposits in the synthesized intercellular matrix were scarce and hardly visible.

**Figure 2:**
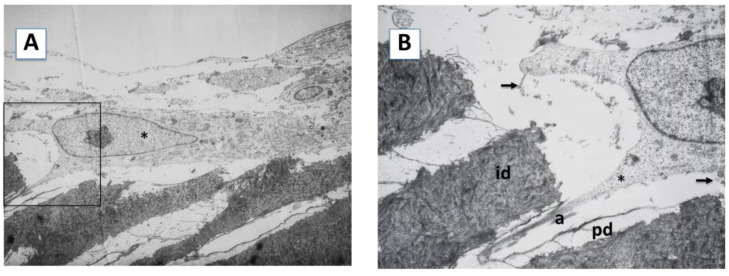

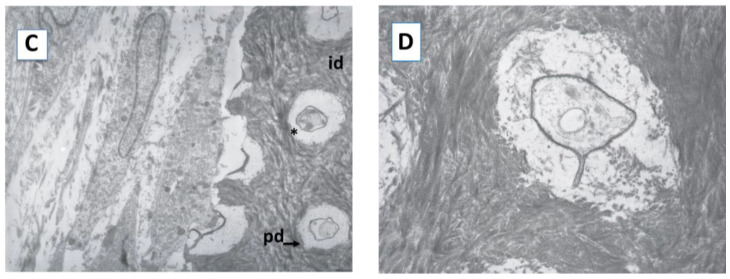
TEM analysis of hDPSCs cultured on the surface A (SA) of the scaffold. (**A**) Odontoblastic-like cell (asterisk) placed on the dentin surface with the tubules perpendicular to the scaffold surface, emitting a process within a dentinal tubule. 1500×. (**B**) Close-up of the area delimited by the square in (**A**). In the cellular process (asterisk), actin microfilaments are observed (a). The cell secrets an extracellular matrix rich in collagen fibers (arrows). Intertubular (id) and peritubular (pd) dentin. 4000×. (**C**) The secretion of a collagen-rich matrix (arrow) is observed in the wall of the dentinal tubule (asterisk) in relation to a process of the hDPSC. Intertubular (id) and peritubular (pd) dentin. 8000×. (**D**) high magnification insert showing the details of a cellular process. 22,000×.

Transmission electron microscopy (TEM) analysis of the samples confirmed and extended these findings. Odontoblast-like cells were found extending processes within the dentinal tubules, on the dentinal surface of the scaffolds. Inside these neo-processes, large amounts of actin microfilaments were observed, which could be participating in the anchoring of the cells to the dentin matrix (Figure 2, panel A). Differences in dentin composition were also observed. As shown in Figure 2, panel B, the intertubular dentin was more electron-dense and contained more collagen fibers than peritubular dentin, which was less electron-dense and contained fewer collagen fibers. Cells cultured on SA showed a large irregular cytoplasm, a large nucleus with non-condensed chromatin, well-developed rough endoplasmic reticulum (RER), and dense bundles of actin microfilaments in the anchored areas of the dentin surface and at the sites of intercellular junctions. The characteristic secretory vesicles were observed both in the cytoplasm and in the cellular processes (Figure 2, panel C). Detailed magnification of a cellular process is shown in Figure 2, panel D. 

### 2.2. Cellular Morphology and Extracellular Matrix Characteristics of hDPSCs Cultured on Surface B (SB)

**Figure 3:**
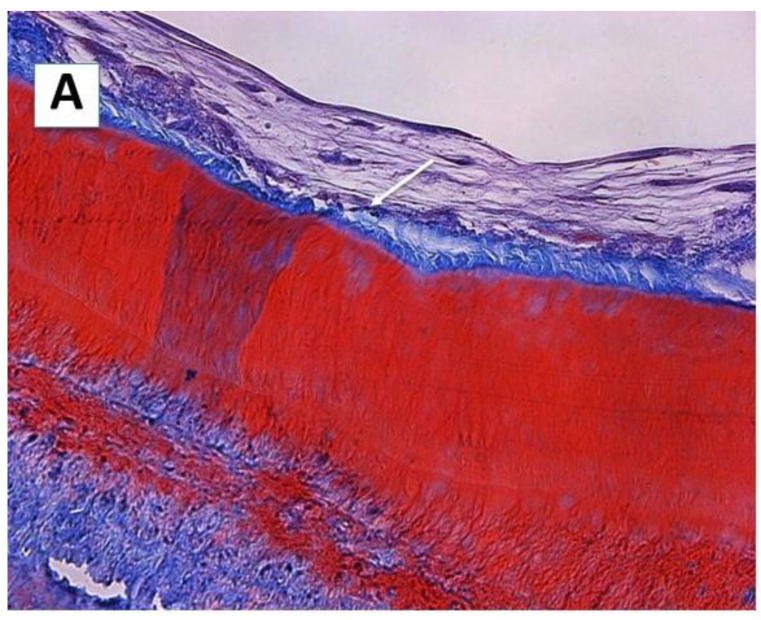

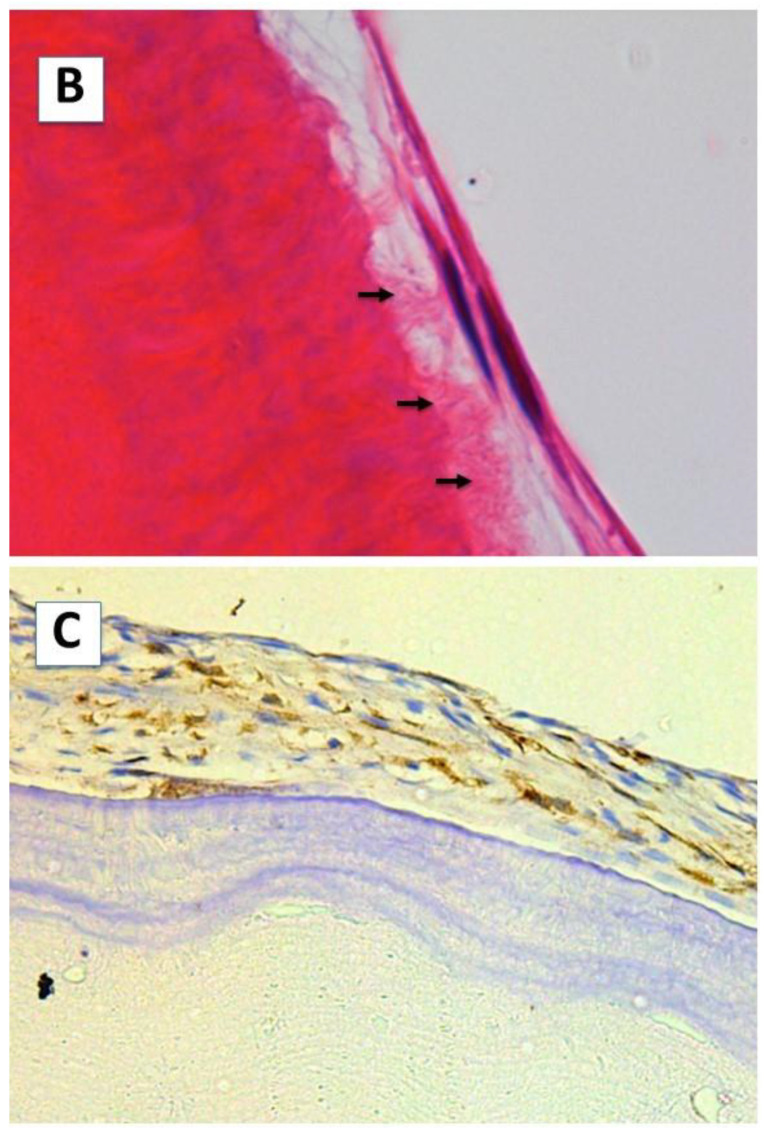
The hDPSCs were cultured on the surface B (SB) of the scaffold for 6 weeks and processed by light microscopy. (**A**): Collagen fibers (arrow) oriented perpendicular to the SB (Masson’s trichrome staining, 40×). (**B**): The hDPSCs formed flattened cell layers, which produced a matrix rich in collagen fibers (arrows) that were preferentially arranged perpendicular to the tooth root surface. Hematoxylin–eosin staining (100×). (**C**): An immunohistochemical study of DSPP protein showed intracellular deposits of DSPP in the hDPSCs and in the newly synthesized intercellular matrix (40×).

The hDPSCs actively proliferated on the cementum surface of the scaffolds. These cells synthesized abundant amounts of extracellular matrix, consisting of dense fibrous connective tissue with thick bundles of collagen fibers (Figure 3, panel A). The density of collagen fibers was higher in proximity to the cementum surface, where thick collagen fiber bundles arranged perpendicular to the cementum surface were observed (Figure 3, panel B). Some DSPP deposits were also observed, not only in the cytoplasm of these cells, but also in the newly formed matrix (Figure 3, panel C). Cells formed more cohesive layers than on surface A, with a denser extracellular matrix and visible DSPP deposits.

On this surface, the hDPSCs acquired a flattened morphology, forming well-organized layers of 3–6 cells surrounded by a newly formed extracellular matrix. The extracellular matrix that was located between the cell layers was looser than that located on the cementum surface (Figure 3, panel A).

**Figure 4:**
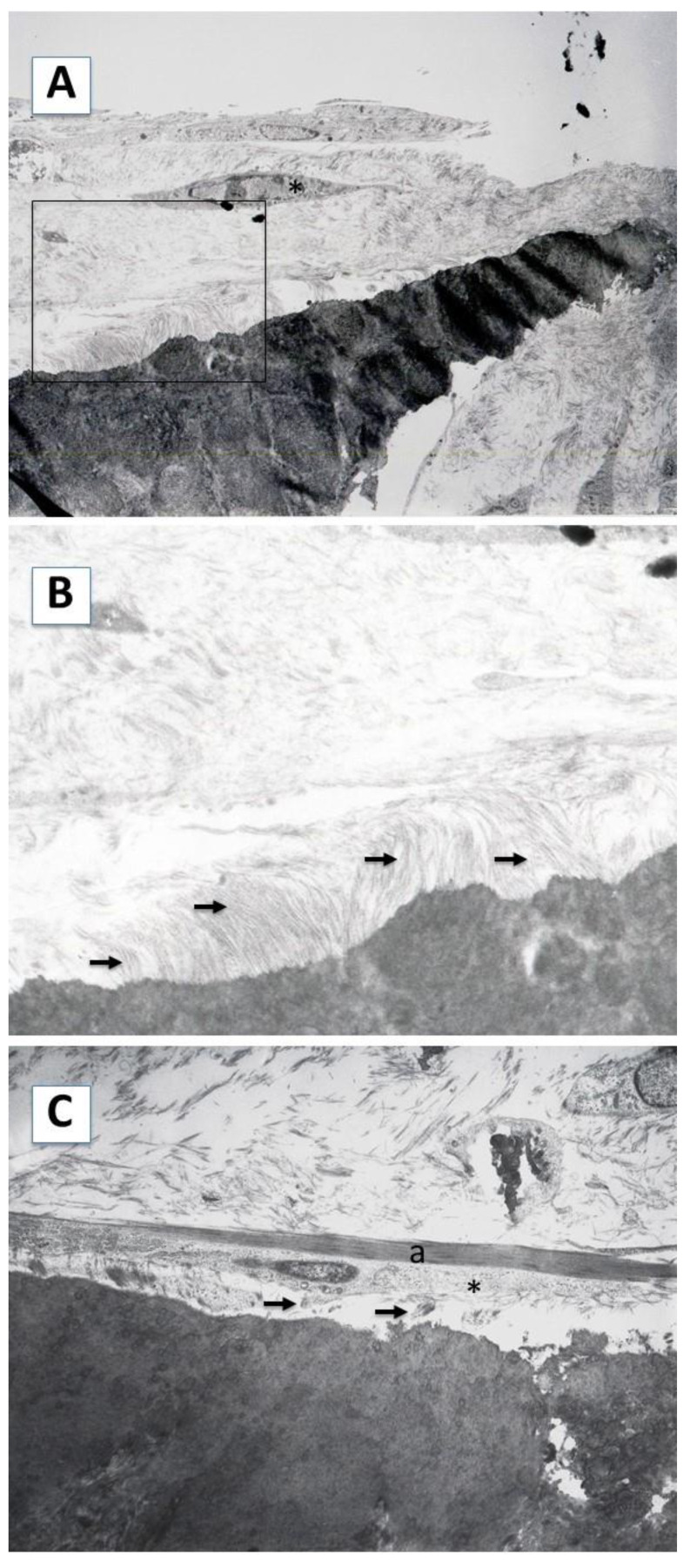
TEM analysis of the hDPSCs cultured on the surface B (SB) of the scaffold. (**A**) The hDPSCs formed layers of flattened cells (asterisk), with abundant production of intercellular matrix. 1500×. (**B**) Close-up of the area delimited by the square in (**A**). The collagen fibers synthesized on the cementum are arranged perpendicular to the surface of the tooth root (arrows). 8000×. (**C**) The hDPSCs adopted a flattened appearance (asterisk). Thick accumulations of actin microfilaments (a) were observed in these cells, as well as the secretion of an extracellular matrix rich in collagen fibers (arrows). 2500×.

The ultrastructural study of the samples confirmed and expanded these findings. Concerning the newly formed extracellular matrix, we observed a high density of collagen fibers (Figure 4, panel A). These fibers formed bundles that were irregularly arranged throughout the matrix, except in the proximity of the cementum surface, where thicker bundles were observed (Figure 4, panel B), arranged perpendicular to the scaffold surface. In addition, the insertion of these fibers into the surface of the scaffold was evident. Regarding the cells, a characteristic secretory phenotype was evidenced by the presence of a well-developed RER, abundant secretory vesicles, mitochondria, and glycogen deposits (Figure 4, panel C).

## 3. Discussion

The periodontium is a functional unit formed by three structures that share the same embryological origin: cementum, periodontal ligament, and the alveolar bone. The collagen fibers of the periodontal ligament are inserted into one end of the cementum, and at the other end, in the alveolar bone, constituting a gomphosis-type joint known as the dentoalveolar joint. This joint holds the tooth in place and allows it to withstand chewing forces. Furthermore, it is a dynamic structure that evolves throughout the life of the tooth in such a way that there is, on the one hand, a remodeling of the periodontal ligament fibers and bone tissue, and on the other, selective appositional growth of the cementum [11].

The accumulation of different bacteria of various genera including Aggregatibacter, Porphyromonas, Tannerella, or Treponema, as well as their lipopolysaccharides (LPS), causes an exacerbated response of the immune system that causes the destruction of the tissues that make up the insertion periodontium and the consequent tooth loss [33,34]. Antibiotic and antiseptic procedures, when applied in the early stages of the disease, are usually successful. However, they are not effective in some patients, either due to the advanced stage of the disease or for other reasons, such as genetic factors or the presence of strains that are highly resistant to treatment, requiring the regeneration of damaged tissues. In this sense, different biomaterials have been tested, including ceramic materials (hydroxyapatite or tricalcium phosphate), synthetic polymers (polyglycolic acid, polylactic acid, or polycaprolactone), and natural ones (collagen, fibrin, albumin, hyaluronic acid, chitosan or alginate, among others) [35]. These filler materials have been used alone or in combination with different biochemical factors, such as the platelet-derived growth factor, fibroblast growth factor, insulin growth factors, transforming growth factor, or bone morphogenetic proteins, as well as many others [35], with varying success. 

The complexity and plasticity of the periodontium have promoted different complementary strategies for the use of these elements, such as the stimulation of dental mesenchymal stem cells for the regeneration of the dentoalveolar joint [24]. There is experimental evidence that demonstrates the ability of different stem cells to regenerate periodontium tissues, including cementum. These cells include periodontal ligament stem cells (PLSCs), apical derived stem cells (ADSCs), and dental follicle stem cells (DFSCs). Some authors suggest that the hDPSCs should be able to differentiate into cementoblasts. The first cementum is formed at the root of the tooth in close contact with the first mineralized dentin, in the proximity of the odontoblasts, and, therefore, the developing dental pulp. Subsequently, ectomesenchymal cells arrive that can differentiate into cementoblasts and are responsible for the appositional growth of cementum. Therefore, it is reasonable to think that the hDPSCs might have the ability to differentiate into cementoid matrix-secreting cells. This hypothesis is in line with our findings. The hDPSCs cultured on SB from acellular tooth scaffolds secreted a compatible cementoid matrix, which includes characteristic perpendicular collagen fibers inserted into the scaffold surface that adopt the characteristic arrangement of the periodontal ligament–cementum interphase [35,36]. These cells acquired a morphology compatible with the secretory cells of the cementoid matrix, consisting of large cells, some elongated with abundant rough endoplasmic reticulum and an extensive Golgi apparatus [37]. 

The study of cells cultured on the SA showed differences in the deposit of the extracellular matrix and its cell morphology. In accordance with previously published data by our research group, these cells acquired an odontoblastic phenotype, with the development of characteristic odontoblastic processes and dentin deposition [33]. It is noteworthy that the only differential element between the two surfaces of the scaffold was the accessibility to the dentinal tubules. Their presence was sufficient for the induction of the odontoblastic phenotype, whereas their absence on the SB resulted in the differentiation into secretory cells and the synthesis of a cementoid-like matrix. These findings reinforce the importance of microtopography in cell differentiation and also demonstrate that, at least in our experimental model, it is not necessary to use biochemical stimuli to induce morphological changes, since the hDPSCs can secrete these factors in a paracrine or autocrine manner.

The factors involved in the regeneration and maintenance of the cementoid matrix continue to be investigated because their knowledge is of vital importance to induce the regeneration of this component of the periodontium. Thus, there are research groups that have demonstrated the role of the DSPP in these processes. Although its role in dentinogenesis is well known, expression of this protein has also been demonstrated in bone, cellular cementum, and several non-mineralized tissues [38,39]. DSPP deficiency has been correlated with severe loss of the alveolar bone, as well as a marked reduction in cementum deposition in transgenic mice [39]. In principle, these deficiencies could be due to defects in the DSPP cleavage of the DSP-G (NH-terminal) and DSP (COOH-terminal) fragments [39]. Taking into account the differences found between animal models developed in rodents and other species, including humans, we aimed to study the expression of this dentinogenesis regulator in our model. We had already demonstrated the importance of DSPP in dentinogenesis using the same experimental model [25]. The findings presented in this work demonstrate the presence of DSPP both in the cytoplasm of the secretory cells and in the formation of deposits in the newly formed cementoid matrix, which reinforce the hypothesis of the involvement of DSPP in the biology of cementum, in addition to its role in the control of dentinogenesis.

In summary, the results presented here point to the potential of the hDPSCs to differentiate in vitro into cells that secrete a cementoid-like matrix, in response to physical stimuli related to the microtopography of the surfaces they come into contact with, which would be a potentially useful cellular element for periodontal regeneration. Likewise, the importance of DSPP in the synthesis and maintenance of tissues other than dentin, such as cement, is highlighted.

## 4. Materials and Methods

### 4.1. Cell Culture

The study was conducted in accordance with the Declaration of Helsinki and applicable local regulatory requirements and laws. All procedures were approved by the Ethics Committee of the University of Valencia (Spain) and all donors provided informed consent. The hDPSCs were isolated as previously described [25]. Briefly, dental pulp was gently removed from human third molars under aseptic conditions using cow horn forceps with a small excavator, and immersed in culture tubes filled with cell culture medium. The specimens were then divided into small pieces using a bistoury blade, immersed in Hank’s balanced salt solution (HBSS), and incubated for 2 h at 37 °C in an atmosphere of 5% CO_2_ and 95% air. The supernatant was removed, 0.1% type I collagenase and dispase (Sigma-Aldrich, Madrid, Spain) in filter-sterilized HBSS were added to the pellet, and the mixture was incubated for 15 min, followed by centrifugation at 1500 rpm for 10 min. The supernatant was removed and the cells were plated in 25-cm^2^ flasks in alpha Minimum Essential Medium (αMEM) culture medium (Sigma-Aldrich) containing 10% fetal calf serum (FCS) (Sigma-Aldrich), penicillin/streptomycin, amphotericin B, 2 mM of L-glutamine, and 100 µM of ascorbic acid (Sigma-Aldrich). The medium was replaced every 3–4 days. Once the cells reached 90% confluence, flow cytometry was performed.

### 4.2. Flow Cytometric Characterization of hDPSCs

The hDPSCs were characterized using a cytometer equipped with a 488-nm Argon laser and a 635-nm red diode laser, as previously described [25]. Experimental data were analyzed using CellQuest software (Becton Dickinson, Madrid, Spain). To exclude cell debris, samples were gated based on their light-scattering properties in the side- and forward-scattered light modes. We recorded 10,000 events per sample within this gate using the medium setting for sample flow rate. The following markers were evaluated: CD29 (Alexa Fluor^®^ 488), CD31 (PE/Cy7), CD44 (PE/Cy5), CD45 (Pacific Blue™), CD105 (APC), and CD146 (PE). Almost 98% of the cells analyzed were positive for CD29, CD44, CD105, and CD146, but negative for CD31 and CD45.

### 4.3. Scaffold Preparation and Study Design

The scaffolds were generated using extracted teeth, as previously reported [25]. Non-endodontic teeth were used in this study. All specimens used in this study had an intact pulp cavity, were free of caries, and showed good crown and apex preservation. Six incisors, two premolars, and seven molars were used. The study was conducted in accordance with the Declaration of Helsinki and applicable local regulatory requirements and laws. All procedures were approved by the Ethics Committee of the University of Valencia (Spain) and all donors provided informed consent. After extraction, specimens were stored at 4 °C in Dulbecco’s phosphate buffered saline (DPBS) supplemented with a solution consisting of 10% antibiotics and fungizone. Teeth were then washed with chlorhexidine and an ultrasound apparatus (Cavitron; Satelec, Cedex, France) and a 7/8 Gracey curette were then used to remove the periodontal ligament, dental calculus, and bacterial biofilm.

A cross-section was made up for the cementoenamel junction using a diamond disk to separate the dental crown and root. The apex was then removed, a diamond bur was used to get access to the pulp chamber, and the pulp tissue was removed using barbed broaches. Endodontic files of different diameters were used to clean and expand the root canal. A similar process was used to completely remove the pulp tissue and predentin covering the pulp chamber. Then, 0.12% chlorhexidine solution was used to clean the dentin surface.

**Figure 5:**
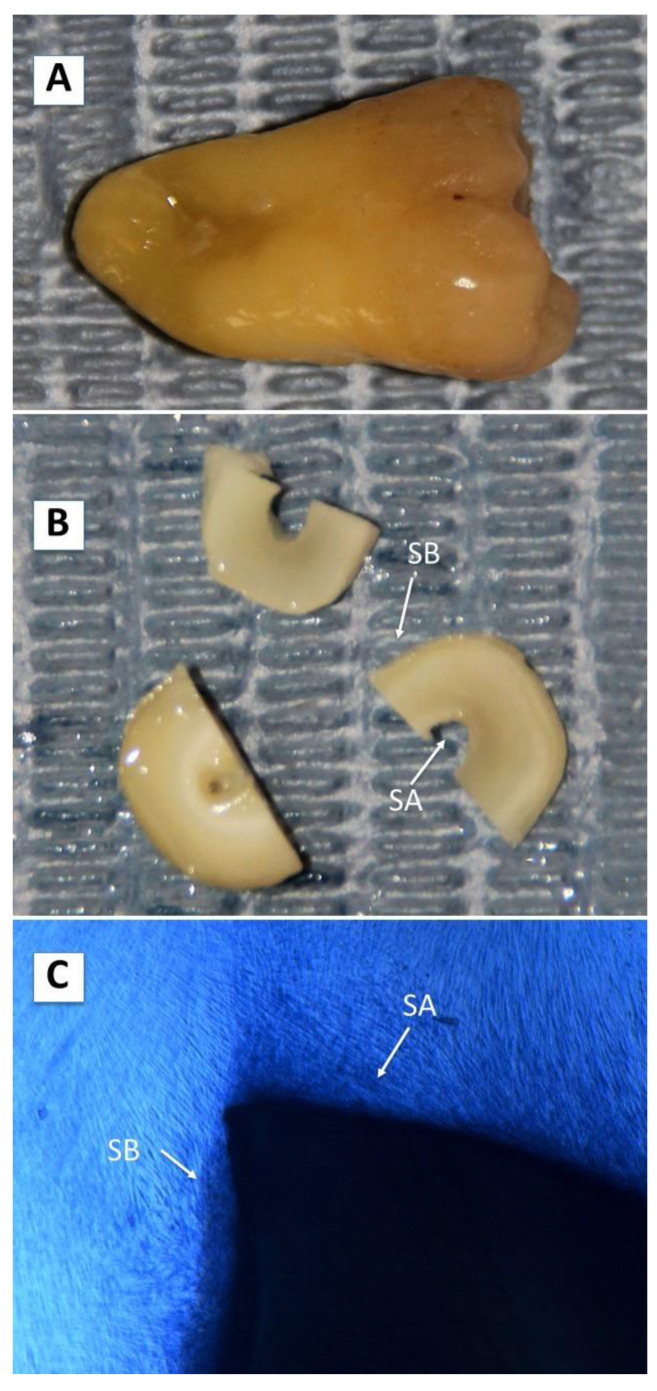
Dentin acellular scaffold preparation. Extracted specimens were washed with chlorhexidine solution and periodontal bacterial biofilm and dental calculus were removed (panel (**A**)). Cross-sectioning to the cement-enamel junction was performed and the apex was removed. Pulp tissue and pre-dentin were completely removed and sections of teeth 1.5-mm thick were obtained and cut into two to three fragments (panel (**B**)). Three different surfaces were considered: surface A (SA: dentin), surface B (SB: cementum). Representative panoramic view of cells grown on SA and SB is shown in panel (**C**) (phase contrast microscopy images, 40 ×).

A diamond disk was used to obtain 1.5-mm thick cross-sections of the roots of the processed teeth, which were then cut into two or three sections (Figure 5, panels A–C). Specimens were washed for 1 h at room temperature with chlorhexidine solution, and then exhaustively washed five times with DPBS supplemented with antibiotics and an antifungal agent at 4 °C for up to 72 h.

The hDPSCs were cultured on scaffolds, in contact with the dentin or cementum surface, as follows. Sections of the roots were placed in 24-well plates and 5,000 cells were seeded in each well, resuspended in 1.5 mL of culture medium, and cultured on the scaffolds for 6 weeks. Every 2 weeks a group of samples was processed for optical and electron microscopy. According to the surface of the tooth root with which the cell contacted, 2 experimental groups were considered for a representative panoramic view of these surfaces (as shown in Figure 5C): cells cultured on surface A (SA: dentinal tubules perpendicular or oblique to the scaffold surface of the accessible tubules) and cells cultured on surface B (SB: cementum surface).

All experiments were performed using 6 replicates with the hDPSCs isolated from the three different donors.

### 4.4. Light Microscopy: Determination of Dentin Sialoprotein Expression

Specimens were washed with DPBS and fixed in 4% buffered formaldehyde solution for 3 h at 4 °C. Decalcification of the scaffolds was performed by incubation in a solution containing 90% Osteosoft (Merck KGaA, Darmstadt, Germany) and 10% formaldehyde solution for up to 21 days. Samples were then embedded in paraffin, cut into 5-µm sections, and stained with hematoxylin and eosin and Masson’s trichrome according to standard protocols.

DSSP expression was evaluated by immunohistochemistry using a mouse anti-human antibody (LFMb-21, dilution 1:50; Santa Cruz Biotechnology, Santa Cruz, CA, USA) according to the manufacturer’s instructions. Sections were deparaffinized and rehydrated using a graded ethanol series, rinsed with distilled water, and treated successively with 0.3% H_2_O_2_ and 10% normal horse serum to block endogenous peroxidase and nonspecific binding, respectively. The Envision amplification system (Envision System + labeled polymer-HRP anti-mouse; Dako, Carpinteria, CA, USA) was used, followed by development with 3,3′-diaminobenzidine (Dako) as chromogen according to the manufacturer’s instructions, which produced brown staining in immunoreactive structures. Sections were finally counterstained with Mayer’s hematoxylin (Sigma-Aldrich).

### 4.5. Transmission Electron Microscopy

The hDPSCs were cultured on scaffolds as described above, washed with DPBS, and fixed in 2.5% glutaraldehyde solution for 4 h at 4 °C. The samples were then decalcified in 4% EDTA for up to 21 days and washed with a solution containing 9% Millonig buffer, 9% calcium chloride, and 1% glucose (Sigma-Aldrich). After postfixation in 2% osmium tetroxide solution, samples were embedded in Epon 812 (TAAB Laboratories Equipment Ltd., Aldermaston, UK) and processed for transmission electron microscopy (TEM), as previously reported [39]. A JEM 1010 electron microscope (JEOL, Tokyo, Japan) operated at 60 kV was used to obtain ultrastructural images.

## Data Availability

Not applicable.
